# Deep sequencing of hepatitis B surface antigen gene in the preserved umbilical cords in immunoprophylaxis failure against mother-to-child HBV transmission

**DOI:** 10.1186/s12879-019-4624-9

**Published:** 2019-11-21

**Authors:** Haruki Komatsu, Ayano Inui, Yasuto Suzuki, Masaya Sugiyama, Tomoo Fujisawa

**Affiliations:** 1 Department of Pediatrics, Toho University, Sakura Medical Center, 564-1 Shimoshizu Sakura, Chiba, 285-8741 Japan; 2 Department of Pediatric Hepatology and Gastroenterology, Eastern Yokohama Hospital, Kanagawa, Japan; 3 Department of Pediatrics, Kushiro Red Cross Hospital, Hokkaido, Japan; 4 Research Center for Hepatitis and Immunology, National Center for Global Health and Medicine, Chiba, Japan

**Keywords:** Hepatitis B virus, Immunoprophylaxis, Umbilical cord, Nails, Mutant, Variant, Phylogenetic tree analysis, Sequencing

## Abstract

**Background:**

Vaccine escape mutants (VEMs) are one of the causes of breakthrough infections in the mother-to-child transmission of hepatitis B virus (HBV). We hypothesized that VEMs existing as minor populations in the maternal blood are associated with breakthrough infections in children. We sought to determine whether VEMs exist as minor populations in the preserved umbilical cords of children with breakthrough infections.

**Case presentation:**

Two families (Family 1: three children, Family 2: two children) were enrolled. Despite immunoprophylaxis, a breakthrough infection occurred in two Family 1 children and two Family 2 children. Preserved umbilical cords, serum, and nails were used for the HBV DNA analysis. To detect VEMs, we performed direct and deep sequencing of hepatitis B surface antigen gene. The direct sequencing showed that there were no VEMs in the serum of the children or mother of Family 1 and family 2, but it identified a G145A mutant in the nails of the mother of Family 2. In Family 1, deep sequencing detected a T143S mutant as a minor population (1.7–2.0%) in the umbilical cords and serum of all three children and in the serum of the mother. A T126A mutant was also detected in the umbilical cord (9.2%) and serum (7.0%) of the first-born child of Family 1. In Family 2, the deep sequencing showed no VEMs in the umbilical cords, but it detected D144A (2.5%) and G145A (11.2%) mutants in the serum of the 2nd-born child.

**Conclusions:**

VEMs were present as minor populations in the preserved umbilical cords of children with breakthrough infections. The VEMs did not become major populations after the breakthrough infections. The evolution of VEMs from a minor form to a major form might not be a prerequisite for breakthrough infections in mother-to-child transmission.

## Background

Hepatitis B virus (HBV) infection is one of the main causes of liver cirrhosis and hepatocellular carcinoma. According to the World Health Organization, an estimated 257 million people worldwide are living with HBV infection [1]. Contact with blood or body fluids from patients with HBV can transmit the infection to other persons [2]. Although the introduction of routine immunization programs with a hepatitis B (HB) vaccine has contributed to the marked reduction of HBV infection, the prevalence of HBV carriers is approx. 6% in Asia and Africa [1], where the main transmission route of HBV is mother-to-child transmission (MTCT) [2].

In order to prevent the MTCT of HBV, HB vaccines alone or HB vaccines plus hepatitis B immune globulin (HBIG) are administered to infants after birth [3–5]. This is a post-exposure type of immunoprophylaxis. High efficacy for preventing MTCT has been shown for this immunoprophylaxis. Unfortunately however, MTCT occurs in 1–3% of children born to HBV-carrier mothers despite the use of immunoprophylaxis [6–8] . High levels of maternal viremia, intrauterine infection, and the emergence of vaccine escape mutants (VEMs) are considered causes of breakthrough infections in MTCT [9]. The mechanisms underlying the emergence of VEMs in MTCT are unknown.

The “a” determinant region shared by all HBV genotypes, which spans amino acids 124 to 147 of the HB surface antigen (HBsAg) gene, is the major target of anti-HBs [10]. The mutation of the “a” determinant region protein is associated with the emergence of VEMs [11, 12]. VEMs are rarely detected by Sanger sequencing in the blood of HBV-carrier mothers whose infants are infected with HBV despite immunoprophylaxis [12, 13]. It has thus been postulated that VEMs are usually present as minor populations in the maternal blood [14–16], and that newborn infants are exposed to maternal blood containing VEMs as minor populations during and after delivery. In the typical clinical course of immunoprophylaxis failure due to VEMs, infants are negative for serum HBsAg and positive for serum anti-HBs for several months after birth. However, VEMs could survive and replicate under the immunological pressure induced by HB vaccines. The infants would then become positive for serum HBsAg and negative for serum anti-HBs [11, 17, 18]. Occasionally, HBsAg and anti-HBs co-exist in infants after the re-appearance of HBsAg. The escape mechanism is the so-called “pre-existence theory.”

In the majority of the studies that reported immunoprophylaxis failure, maternal blood was taken and evaluated after delivery [12, 17–19]. Although a few studies evaluated VEMs in maternal blood samples that had been stored before delivery [14, 20], there is only one prior immunoprophylaxis failure study that analyzed maternal blood taken at the delivery [13].

For the determination of whether the pre-existence theory is correct, maternal blood collected at delivery is preferable to that obtained before or after delivery. In Japan, there is a widespread custom from ancient times of preserving the umbilical cord (stump), which spontaneously falls off and dries out, as a memorial of the birth. This traditional custom is a manifestation of the parents’ desire for the happiness and health of their children. By taking advantage of the umbilical cords preserved for this reason, several studies have investigated congenital cytomegalovirus infection [21–23]. A preserved umbilical cord contains the source of HBV infection at delivery. Without an intrauterine infection, a preserved umbilical cord may reflect the HB viral quasi-species of the maternal blood at delivery. It is thus possible that preserved umbilical cords could be useful to test the pre-existence theory in immunoprophylaxis failure due to VEMs.

We conducted the present study to determine whether VEMs are present as minor populations in the preserved umbilical cords of cases of immunoprophylaxis failure. Two families who experienced immunoprophylaxis failure were evaluated. In addition to the preserved umbilical cord, nails were used for the analysis of the HBV genome. Sanger sequencing of the complete HBV genome and the deep sequencing of the HBAg gene including the “a” determinant region were also performed.

## Case presentation

### Family 1

The characteristics of Family 1 are shown in Table 1. The mother was chronically infected with HBV through MTCT. She did not receive immunoprophylaxis at birth. She was positive for HBeAg. She bore three children who were delivered via vaginal. All three children received immunoprophylaxis (HB vaccine plus HBIG) after birth. However, the 1st-born and 2nd-born children became HBV carriers. Although the 1st-born child was negative for HBsAg and positive for anti-HBs (155 mIU/mL) at the age of 2 weeks, she became positive for HBsAg and negative for anti-HBs at the age of 3 months.

**Table 1:** Characteristics of the mother and children in Family 1 and Family 2

Family	Patients	Year of birth	Immuno- prophylaxis	Gender	Serum ALT level	HBV serological status in 2016				Quantification of HBV DNA		
						HBsAg	HBeAg	Anti-HBs	Anti-HBc	Sample	Year of sampling	HBV DNA (log copies/mL)
1	Mother	1985	Not done	Female	17	Pos	Pos	Neg	Pos	serum	2016	9.0
	1st-born child	2004	Failure	Female	22	Pos	Pos	Neg	Pos	Dried umbilical cord	2004	3.7
										serum	2016	7.8
	2nd-born child	2007	Failure	Male	12	Pos	Pos	Neg	Pos	Dried umbilical cord	2007	4.7
										serum	2016	8.3
	3rd-born child	2015	Success	Female	11	Neg	Neg	Pos	Neg	Dried umbilical cord	2015	3.8
2	Mother	1988	Not done	Female	28	Pos	Pos	Neg	Pos	Nail	2016	3.8
	1st-born child	2013	Failure	Female	134	Pos	Pos	Neg	Pos	Dried umbilical cord	2013	4.5
										serum	2016	8.6
										Nail	2016	4.9
	2nd-bornchild	2014	Failure	Male	129	Pos	Pos	Neg	Pos	Dried umbilical cord	2014	3.8
										serum	2016	7.6
										Nail	2016	3.8

Pos: positive Neg: negative

The 2nd-born child was already positive for HBsAg and negative for anti-HBs at the age of 2 months. Fortunately, the immunoprophylaxis was successful in the 3rd-born child, who was negative for HBsAg and positive for anti-HBs at the age of 12 months. Moreover, serum HBV DNA was undetected by real-time polymerase chain reaction (PCR) in the 3rd-born child at the age of 24 months. The mother did not receive any antiviral treatment during pregnancy.

### Family 2

The characteristics of Family 2 are shown in Table 1. The mother was an HBV carrier (HBeAg: positive, serum HBV DNA in April 2016: 9.0 log copies/mL). The source of her HBV infection was unknown. She had two children who were born via vaginal delivery. Both children received immunoprophylaxis (HB vaccine plus HBIG) after birth. The 1st-born child was positive for HBsAg and negative for anti-HBs at the age of 1 month. The 1st-born child became positive for anti-HBs at the age of 6 months, but the level of anti-HBs was low (22 mIU/mL). The 1st-born child became negative for anti-HBs again at the age of 7 months. The 2nd-born child was negative for HBsAg and positive for anti-HBs (342 mIU/mL) at the age of 9 months. However, the 2nd-born child became positive for HBsAg and negative for anti-HBs at the age of 11 months. Although the mother did not receive any antivirals during pregnancy, she started to receive antiviral treatment with tenofovir disoproxil fumarate (TDF) in April 2016, and her level of serum HBV DNA then decreased to 3.8 log copies/mL in July 2016. Serum HBV DNA was not detected in the mother by conventional PCR in November 2016.

#### HBV DNA extraction and the quantification of HBV

Serum HBV DNA was extracted from 200 μL of serum using a QIAamp DNA Blood Mini kit (Qiagen, Hilden, Germany). Dried umbilical cord HBV DNA was extracted from 20 to 30 mg of dried umbilical cord using a QIAamp DNA Mini kit (Qiagen). Nail HBV DNA was extracted using a DNA Extractor FM kit (Wako Pure Chemical Industries, Osaka, Japan). Serum HBV DNA was measured by the COBAS TaqMan HBV DNA test ver. 2.0 (Roche Diagnostics, Tokyo). An in-house real-time assay was used for the quantification of HBV DNA from umbilical cord and nails [24]. Sequencing of the complete HBV genome was performed on the basis of the previous study [25].

#### Phylogenetic tree analysis

The DNA sequences were aligned by using the Genetyx software program (ver. 11; Software Development Co., Tokyo). The phylogenetic tree was constructed by the neighbor-joining method [26], with pairwise distances being estimated by the Kimura two-parameter method. The reliability of the phylogenetic tree thus obtained was assessed with 1000 bootstrap replicates [27]. The evolutionary distances were computed using the Maximum Composite Likelihood method [28]. These analyses were performed with the MEGA 7.0.21 software program (http://www.megasoftware.net/) [29]. The nucleotide sequence data reported in this paper appear in the DDBJ/EMBL/GenBank nucleotide sequence databases with the accession numbers LC279268–76, LC318702, and LC318703.

#### Deep sequencing for the HBsAg gene

The HBsAg gene, including the major hydrophilic region (amino acids 99–169 of HBsAg) was amplified (432 bp) by nested PCR as described [20, 30]. All samples were amplified with a proofreading enzyme (high-fidelity enzyme; PrimeSTAR MAX DNA polymerase, TaKaRa Bio, Shiga, Japan) in the PCR. The PCR amplicons of each sample were pooled and subjected to deep sequencing. Paired-end sequencing was performed on an Illumina MiSeq sequencing system (Illumina, San Diego, CA) with a MiSeq Reagent Kit Nano ver. 2 (Illumina). Initially, raw sequences were processed using the quality-filtering software CASAVA ver. 1.8.2 (Illumina). Sequence reads were aligned with the HBV reference sequence of HBV genotype B (GenBank/EMBL accession no. AB010291) and genotype C (GenBank/EMBL acc. no. AB033550) using the alignment tool BWA (0.7.15) (https://sourceforge.net/projects/bio-bwa/files). The accuracy of the ultra-deep sequencing in this platform for detecting low-level viral mutations was considered to be > 1%.

#### Locked nucleic acid-based probe real-time PCR for G145A mutant

The two sets of paired primers used for the real-time PCR were as follows: forward: TCCTGCTCAAGGAACCTCTA (nt 532–551), reverse: CAGGATGATGGGATGGGAATAC (nt 600–621); (amplicon size, 90 bp). The following locked nucleic acid (LNA)-based probes (nt 582–591) designed for discrimination between the glycine to alanine at the 145th amino acid of HBsAg (G145A mutant: nucleotide position at 588: G → C) and the wild-type were used for the real-time PCR. Probe-GG (wild-type sequence): 5′-FAM- TT**TCC**G**T**C**C**G-IBFQ-3′, and Probe-GC (mutant-type sequence): 5′-HEX-TT**TGC**G**T**C**C**G -IBFQ-3′ (Integrated DNA Technologies, Coralville, IA, USA). The LNA nucleotides are shown in bold and underlined letters. The lower detection limit was > 1000 copies/mL. The LNA-based real-time PCR can detect subpopulations at 1% of the HBV population. All assays were carried out in triplicate with negative control samples. The wild-type and G145A mutant-type DNA extracted from serum were quantified according to the recombinant plasmid wild-type controls and the G145A mutant-type (nucleotide position at 588: G → C) controls, respectively. The number of the nucleotide position was based on the genotype C (GenBank/EMBL acc. no. AB033550).

#### HBV DNA levels in the serum, umbilical cord, and nails

In Family 1, serum samples were taken from the 1st-born child, 2nd-born child, and mother. Individual dried umbilical cord samples were provided from all three children in the family. In Family 2, serum samples from both of the children and individual dried umbilical cord samples for both children were also provided. Because HBV DNA extracted from the serum of the Family 2 mother was not detected by conventional PCR, her fingernails, which were considered to be a reservoir of HBV DNA [31, 32], were used for the Sanger sequencing. For a comparison with the nails of the mother, nail samples from the two children were also used for the Sanger and deep sequencing. Except for the dried umbilical cord samples, all of the samples were taken in 2016. The HBV DNA levels of the serum, umbilical cord samples, and nail samples (concentration of total DNA extracted from nails: mother; 50 μg/mL, 1st-born child; 23 μg/mL, 2nd-born child;17 μg/mL, β actin DNA levels in nails: mother; 8.5 log copies/mL, 1st-born child; 8.0 log copies/m, 2nd-born child; 8.0 log copies/mL) are shown in Table 1. All of the HBV carriers showed high viral loads (≥7.6 log copies/mL) in their blood. The HBV DNA level of the five umbilical cord samples ranged from 3.7 log copies/mL to 4.7 log copies/mL (median 3.8 log copies/mL). The HBV DNA level of the nail samples was 3.8 log copies/mL in the mother of Family 2, 4.9 log copies/mL in her 1st-born child, and 3.8 log copies/mL in her 2nd-born child.

#### The full-length HBV genome and the phylogenetic analysis

**Fig. 1:**
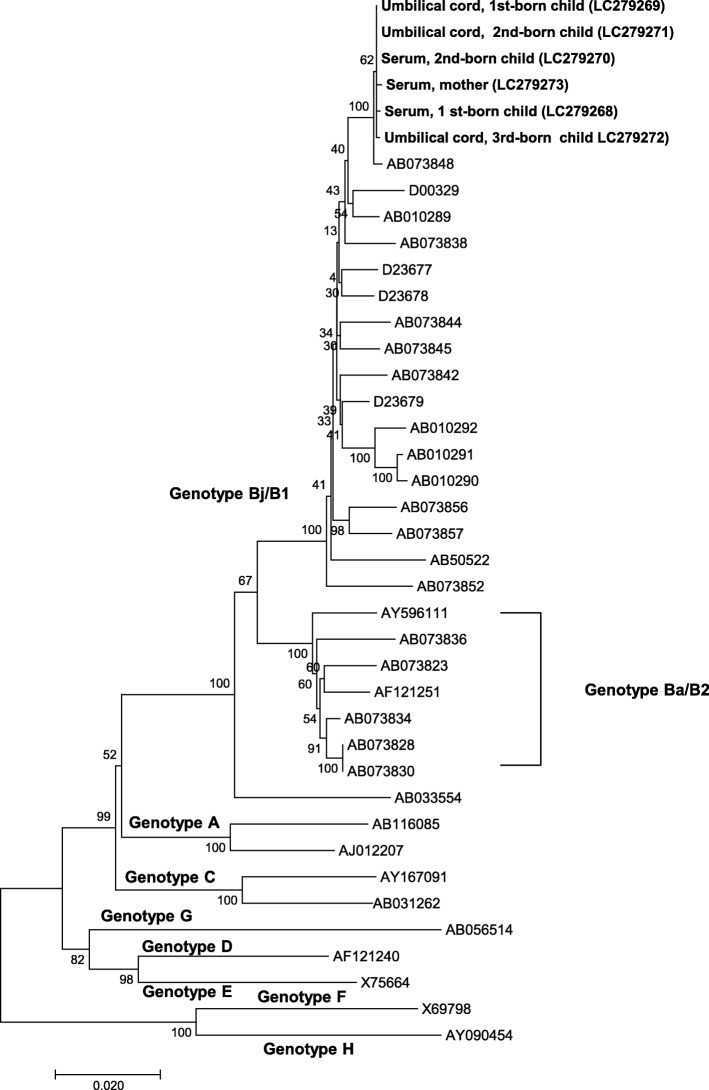
Phylogenetic analysis of Family 1 The percentage of replicate trees in which the associated taxa clustered together in the bootstrap test (1000 replicates) is shown next to the branches. The tree is drawn to scale, with branch lengths in the same units as those of the evolutionary distances used to infer the phylogenetic tree. The analysis involved 34 nucleotide sequences

**Fig. 2:**
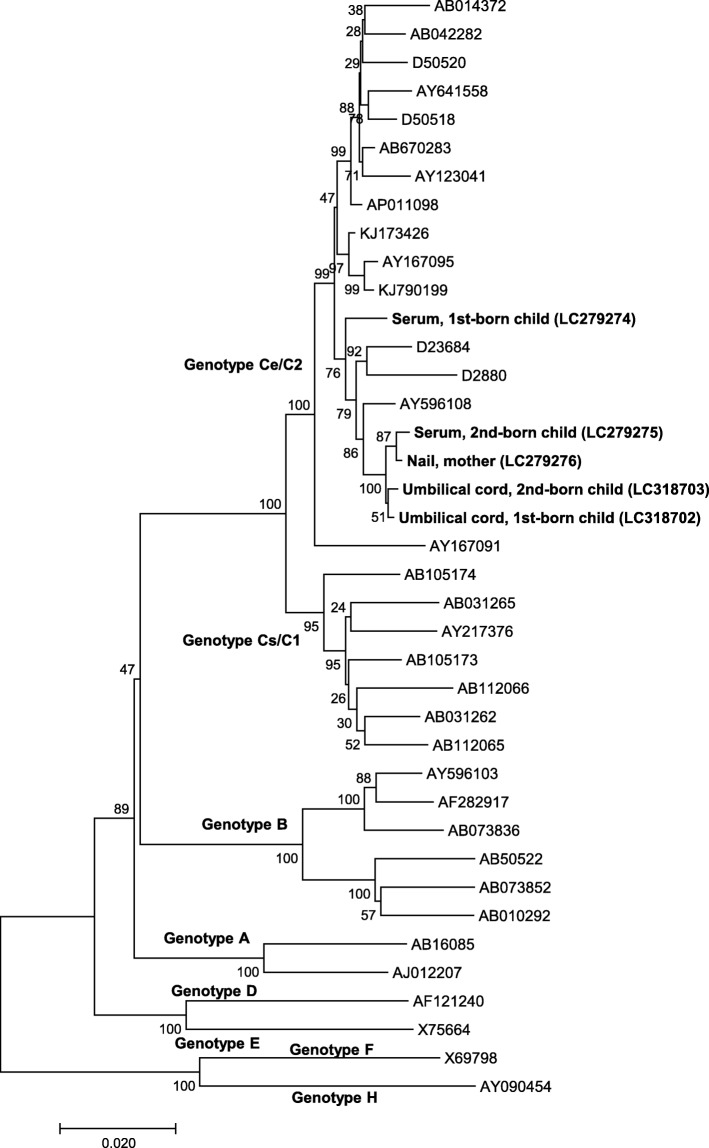
Phylogenetic analysis of Family 2 The percentage of replicate trees in which the associated taxa clustered together in the bootstrap test (1000 replicates) is shown next to the branches. The tree is drawn to scale, with branch lengths in the same units as those of the evolutionary distances used to infer the phylogenetic tree. The analysis involved 34 nucleotide sequences

In Family 1, the complete HBV genome sequence was successfully identified in the three serum samples (mother, 1st-born, and 2nd-born) and three umbilical cord samples. In Family 2, the complete HBV genome sequence was successfully identified in two serum samples (1st-born and 2nd-born), two umbilical cord samples, and one nail sample (mother). The results of the phylogenetic analysis are provided in Figs. 1 and 2. All six of the HBV sequences from the mother and children of Family 1 belonged to HBV genotype Ba/B1 and formed one cluster. Similarly, all five of the HBV sequences from the mother and children of Family 2 belonged to HBV genotype Ce/C2. In Family 2, although the sequences in the serum from the 2nd-born child, the mother’s nail sample, and the umbilical cord samples formed one cluster, the sequence in the serum from the 1st-born child was a little distant from the cluster. These findings suggest that the sequences derived from umbilical cord and nails are useful to evaluate the evolution of the HBV genome sequence.

**Fig. 3:**
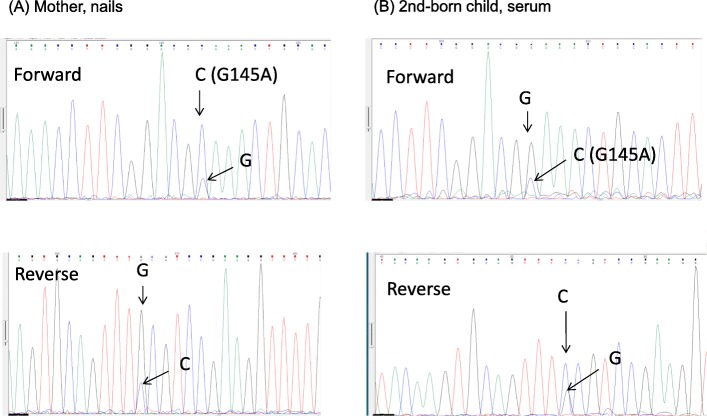
Electropherogram of direct sequencing **(a)** Mother’s nail sample. **(b)** 2nd-born child. The forward and reverse read show two peaks at the nucleotide position 588 of HBs antigen in both the nails and serum. A: In the forward read, the large peak indicates “C” (G145A) and the small peak indicates “G” (wild-type) in the forward read. In the reverse read, the large peak indicates “G” and the small peak indicate “C”. B: In the forward read, the large peak indicates “G” (wild-type) and the small peak indicates “G” (G145A) in the forward read. In the reverse read, the large peak indicates “C” and the small peak indicate “G”

In addition to the phylogenetic tree analysis, we evaluated the amino acid sequence of the “a” determinant region. In Family 1, all of the amino acid sequences of the “a” determinant region were identical. There was no amino acid mutation in the “a” determinant region. In Family 2, a mutation from glycine to alanine at the 145th amino acid of HBsAg gene (nucleotide position at 588: G → C) was detected in the mother’s nail sample. The G145A mutation is shown in Fig. 3. Although the peak of the electropherogram indicates “C” at the nucleotide position 588 of HBsAg gene, a small peak indicating “G” coexists at the mutation (Fig. 3a). The remaining four samples (two umbilical cord samples, two serum samples) showed no mutation in the “a” determinant region. However, a small peak of “C” was observed at the nucleotide position 588 of HBsAg gene in serum of the 2nd-born child in Family 2 (Fig. 3b). The reaming three samples had no small peak of “C” at the nucleotide position 588 of HBsAg gene. These findings suggest that the G145A mutant exists as a minor population in the 2nd-born child.

#### The frequencies of HBsAg gene variants evaluated by deep sequencing

### Family 1

The frequencies of the HBV variants of Family 1 are shown in Table 2. Eleven variants were detected in HBsAg gene by the deep sequencing. Except for the S40 N variant, there was no variant as a major population in HBsAg gene from the umbilical cords and serum. Of the 10 minor variants, four (I110L, S113 T, K122Q, and T143S) were detectable in all of the umbilical cord samples and all serum samples. V47A was also detected in all samples except for the umbilical cord of the 1st-born child. P120S was detected in the serum of the mother and 1st-born child of Family 1. T126A was detected in the umbilical cord and serum of this 1st-born child. G44E, G102C, and Y161F were detected in the serum of the 1st-born, the umbilical cord of the 1st-born, and the umbilical cord of the 3rd-born child, respectively.

**Table 2:** Frequencies of HBV variants analyzed by deep sequencing in Family 1

	2005	2007	2015	2016	2016	2016
HBV variants	1st-born child	2nd-born child	3rd-born child	Mother	1st-born child	2nd-born child
*n* = 11	Umbilical cord	Umbilical cord	Umbilical cord	Serum	Serum	Serum
S40 N	50.0	50.0	50.0	50.0	50.0	50.0
G44E					1.0	
V47A		1.0	1.1	1.0	1.0	1.0
G102C	16.3					
I110L	2.4	2.5	2.6	2.8	2.9	2.7
S113 T	1.0	1.2	1.2	1.3	1.5	1.3
P120S				1.0	2.9	
K122Q	1.8	2.0	2.4	1.9	2.4	1.9
T126A	9.2				7.0	
T143S	1.7	1.7	1.8	2.0	2.0	2.0
Y161F			3.1			

All data in the table are percentages

Interestingly, in Family 1 the frequencies of the individual HBV variants showed similar values in the three umbilical cords and three serum samples. Of the 11 variants, T126A and T143S were located in the “a” determinant region. T126A was detected in the umbilical cord (9.2%) and serum (7.0%) of the 1st-born child, and T143S was detected in all three umbilical cords (1st-born: 1.7%, 2nd-born: 1.7%, 3rd-born: 1.8%) and in three serum samples (mother: 2.0%, 1st-born: 2.0% 2nd-born: 2.0%). T126A [13, 17, 33–35] and T143S [36–39] have been reported as VEMs. These findings indicate that the three children were exposed to the VEMs at delivery. However, our direct and deep sequencing showed that the VEMs did not evolve from a minor form into a major form to compete with anti-HBs induced by immunoprophylaxis.

### Family 2

The frequencies of the HBV variants of Family 2 are shown in Table 3. Thirteen variants were detected in HBsAg gene by the deep sequencing. Of the 13 variants, only T114S was detected as a major population in all of the samples from umbilical cords, serum, and nails. I68V was detected as a minor population in all seven samples. I68T was detected in both the serum and nails of the 1st-born and 2nd-born children. G44E, P56L, F93S, C107Y, L110P, S117 N, T118K, D144A and W167 L were detected in only one sample. Although P56L, I68V, and I68T were detected as a minor population in the three umbilical cord samples, there was no variant in the “a” determinant region of HBsAg gene from the three umbilical cord samples. However, D144A (2.5%) and G145A (11.2%), which were located in the “a” determinant region, were detected as a minor population in the serum of the 2nd-born child. Although the direct sequencing showed that G145A was present as a major population in the mother’s nails, the deep sequencing showed that G145A (34.8%) was present as a minor population in her nails. Both D144A [14, 16, 35, 40–43] and G145A [33, 35, 43, 44] were reported to be VEMs.

**Table 3:** Frequencies of HBV variants analyzed by deep sequencing in Family 2

	2013	2014	2017	2017	2017	2017	2017
HBV variants	1st-born child	2nd-born child	1st-born child	1st-born child	2nd-born child	2nd-born child	Mother
*n* = 13	Umbilical cord	Umbilical cord	Serum	Nails	Serum	Nails	Nails
G44E				1.8			
P56L		6.7					
I68V	49.4	49.2	12.2	11.5	48.1	41.8	49.3
I68T			37.8	38.5	1.5	8.4	
F93S							2.0
C107Y						15.0	
L110P				1.9			
T114S	100	100	100	100	100	100	100
S117 N				2.0			
T118K					23.4		
D144A					2.5		
G145A					11.2		34.8
W167 L						8.3	

All data in the table are percentages

#### The quantification of G145A mutant in family 2

There was thus a discrepancy in the population of G145A mutant from the nails of the mother of Family 2 between the direct sequencing and the deep sequencing. To re-evaluate the populations of G145A mutant in the umbilical cords, serum, and nails of Family 2, we performed an LNA-based probe real-time PCR. Similar to the deep sequencing, the LNA-based probe real-time PCR did not detect G145A mutant in the umbilical cord samples of Family 2. However, G145A mutant was detected in the nails of the 2nd-born child. The levels of wild-type HBV DNA were 3.2 log copies/mL in the mother’s nails, 7.5 log copies/mL in the serum of the 2nd-born child, and 3.7 log copies/mL in the nails of the 2nd-born child. The levels of G145A mutant were 3.8 log copies/mL in the mother’s nails, 7.0 log copies/mL in the serum of the 2nd-born child, and 3.3 log copies/mL in the nails of the 2nd-born child. The quantification of G145A mutant showed that G145A was present as a major population in the nails of the mother and as a minor population in the serum and nails of the 2nd-born child. These findings were consistent with the results of the direct sequencing.

## Discussion and conclusions

To investigate the existence of VEMs as minor populations in the source of HBV infection at delivery, we evaluated the preserved umbilical cord samples of children with an HBV breakthrough infection by performing direct and deep sequencing. This is apparently the first study to evaluate HBV DNA derived from preserved umbilical cord and nail samples by deep sequencing. HBV DNA was detectable in all five of the dried umbilical cord samples, and the full-length sequence of HBV genome was successfully identified in the umbilical cord samples. Because the 1st-born child in Family 1 and the 1st- and 2nd-born children in Family 2 showed the reversion from the positivity of anti-HBs to negativity during the clinical course, it was strongly suspected that the VEMs were associated with the breakthrough infection in these children.

We observed that VEMs were not detectable in the umbilical cord samples by direct sequencing, but the deep sequencing showed that VEMs (T143S) were present as minor populations in all of the umbilical cord samples from Family 1. In addition to T143S, T126A was present as a minor population in the umbilical cord of the 1st-born child of Family 1. Notably, T126A has often been reported as a VEM [13, 17, 33–35], and the combination of T126A plus T143S was often detected in Indonesian children with breakthrough infections [37, 38]. The results of our analyses confirmed that all three children in Family 1 were exposed to VEMs at delivery. However, T126A and T143S were detected as minor populations in serum. Moreover, the frequencies of T126A and T143S showed almost the same levels in the umbilical cord samples and serum after the breakthrough infections.

There are two possibilities to explain these findings. One is the occurrence of reversion from VEMs to wild-type after a breakthrough infection. After VEMs became major populations to break through the immunological pressure of anti-HBs, the VEMs might revert to wild-type at the previous level with the loss of anti-HBs. Although there is no report of the reversion of VEMs to wild-type in immunoprophylactic treatment with an HBIG or HB vaccine, the reversion of nucleos(t) ide analogue (NA)-resistant mutants to wild-type can occur after the cessation of NA therapy [45, 46]. The other possibility is that VEMs can cause a breakthrough infection even if they are minor populations. When the maternal viral load in the blood is high, the viral load of VEMs as minor populations is also high. One of our previous studies demonstrated that four of six children who were chronically infected with HBV due to a breakthrough infection had the G145R mutant had a minor population in their serum [20]. In the animal experiment, chimpanzees were protected from infection of VEMs (G145R) by a recombinant HB vaccine [47]. This finding is inconsistent with the results of the present study. The animal experiment was pre-exposure immunoprophylaxis. On the other hand, the immunoprolhylaxis for mother-to-child transmission of HBV is post-exposure. Additionally, the viral load (10^4.0^ CID50) which was used for inoculation in the animal experiment might be different form that of the two mothers in this study. These differences could have contributed to the inconsistent results. Further studies are necessary to determine whether VEMs cause a breakthrough infection without the evolution from a minor form to a major form.

In contrast to Family 1, neither the direct sequencing nor the deep sequencing could detect any VEMs in the umbilical cord samples from Family 2. However, D144A and G145A mutants were detected by the deep sequencing as minor populations in the serum of the 2nd-born child of Family 2. Although the deep sequencing did not detect the G145A mutant in the nails of the 2nd-born child, the LNA-based probe real-time PCR detected the G145A mutant as a minor population in the nails of the 2nd-born child. The G145A mutant was detected as a major population in the nails of the mother of Family 2.

There are two possibilities to explain why the deep sequencing failed to identify the G145A mutant in the umbilical cord; one possibility is based on the detection limit of the frequencies in deep sequencing. Minor populations below 1% could not be detected by deep sequencing in this study, and the existence of VEMs as minor populations below 1% was thus not identified in this study. To improve the limitation of detection in deep sequencing, tag-linked deep sequencing was developed [48]. This new technique can discriminate 0.1% of viral populations. An earlier study showed that VEMs as minor populations below 1% in a mother became predominant in her children with breakthrough infections [48]. The other possibility is that the G145A mutant was caused by de novo mutation. However, the G145A mutant existed as a major population in the nails of the mother of Family 2, and it is thus plausible that the G145A mutant, which was transmitted from the mother to the 2nd-born child, was present as < 1% of the viral population in the umbilical cord. The 2nd-born child of Family 1 and the 1st-born child of Family 2 were positive for serum HBsAg at the ages of 2 months and 1 month, respectively. The possibility of intrauterine infection should thus not be excluded in both children.

In this study, HBV DNA was detected in the umbilical cord and nail samples. Despite the low viral load in blood due to antiviral treatment, an adequate amount of HBV DNA for conventional sequencing was extracted from the nail samples. Our preliminary data suggest that HBV DNA is detectable for PCR in nails after serum HBV DNA becomes undetectable (data not shown). Umbilical cords and nails are useful for the analysis of HBV genomes. Our phylogenetic analysis revealed that the HBV full-length genome sequences from the umbilical cords, nails, and serum consist of one cluster in Families 1 and 2. Moreover, the deep sequencing revealed that the distribution and frequencies of the detected HBV variants were almost the same among the family members. These findings indicate that the rapid viral evolution of quasi-species does not occur in the children and mothers.

However, this study shows a limitation in the umbilical cord for analysis. The third-born child in family 1 whose umbilical cord was positive for VEM was not infected, whereas the two children in family 2 whose umbilical cords were negative for VEM were infected. This finding might indicate that HBV DNA extracted from umbilical cord could not perfectly reflect the viral quasi-species of blood. Moreover, the evaluation of umbilical cords from children with successful immunoprophylaxis as control might be helpful to clarify whether the pre-existence of VEMs is associated with immunoprophylaxis failure in this study.

All of the family members with chronic HBV infection in this study were positive for HBeAg, with a high viral load and a persistent normal range of serum ALT (except for the ALT levels of the 2nd-born child in Family 2). They are at the so-called immune-tolerant phase (Phase 1) [49]. Because patients with a chronic HBV infection lack robust immunological pressure on HBV in the immune-tolerant phase, the distribution and frequencies of detected HBV variants as minor populations could remain almost the same after the transmission from mothers to children.

We cannot clearly explain the causes of two observations: (1) The reason for the successful immunoprophylaxis in the 3rd-born child of Family 1 despite the exposure to T143S mutant at delivery is not known. The maternal HBV DNA level is the most important predictor for a breakthrough infection in mother-to-child transmission. To prevent the MTCT of HBV, the clinical practice guidelines issued by the European Association for the Study of Liver recommend antiviral prophylaxis with TDF for pregnant women with a high viral load (> 200,000 IU/ml) [49]. We did not have the data of the maternal HBV DNA level before the delivery of the 3rd-born child in Family 1, but the mother remained positive for HBeAg with a high viral load after the delivery of her 3rd-born child. It is likely that the maternal viral load in the blood remained at a high level through the three pregnancies. Therefore, the maternal viral load cannot explain the different outcomes of immunoprophylaxis among the three children in Family 1. The response to an HB vaccine, which is associated with human leukocyte antigen [50], and the timing of the immunoprophylaxis may affect the outcomes of immunoprophylaxis.

(2) The other unexplained observation is the discrepancy in the frequency of G145A in the nail samples between the direct sequencing and deep sequencing. The electropherogram of the direct sequencing showed that the G145A mutant was predominant in the nails. The LNA-based probe real-time PCR results confirmed that the HBV DNA level of G145A was higher than that of the wild-type in the nail samples. The reverse read of the deep sequencing showed G145A mutant at the frequency of 69.6% (not shown). However, there was no forward read including aa145 in the deep sequencing. The frequency of G145A was therefore calculated as 34.8% in the nails. If we had performed long-read deep sequencing, this discrepancy might have been prevented.

In conclusion, the pre-existence of VEMs as minor populations was confirmed in the preserved umbilical cord samples by deep sequencing. However, the pre-existing VEMs did not become major populations after the breakthrough infections. The viral evolution of a minor form to a major form might be not necessary to break through immunoprophylaxis. The nail samples from HBV carriers were useful for the evaluation of viral quasi-species.

## Supplementary information

**Additional file 1: Figure S1.** The specificity of locked nucleic acid-based probe real-time PCR. (A) The wild-type plasmid (DNA level: 9.0 log copies/mL) was used for real-time PCR. The wild-type probe (FAM) detected the wild-type plasmid, but the G145A probe (HEX) did not detect the wild-type plasmid. (B) The G145A plasmid (DNA level: 9.0 log copies/mL) was sued for the real-time PCR. The Wild-type probe (FAM) did not detect the G145A plasmid, but the wild-type probe (HEX) did not detect the G145A-type plasmid. (PPTX 96 kb)

**Additional file 2: Figure S2.** The specificity of locked nucleic acid-based probe real-time PCR. Wild-type plasmid (DNA level: 8.2 log copies/mL) and G145A plasmid (DNA level: 8.2 log copies/mL) were mixed in this study. Population of G145R plasmid: (A) 100%, (B) 50%, (C) 25%, (D) 10%, and (E) 1%. (PPTX 153 kb)

## Data Availability

All data generated or analyzed during this study are included in this published article and its Additional files 1 and 2.

## Abbreviations

HB: Hepatitis B
HBIG: Hepatitis B immune globulin
HBsAg: Hepatitis B surface antigen
HBV: Hepatitis B virus
LNA: Locked nucleic acid
MTCT: Mother-to-child transmission
TDF: Tenofovir disoproxil fumarate
VEM: Vaccine escape mutant
