# Supplementary material for: Deep sequencing of hepatitis B surface antigen gene in the preserved umbilical cords in immunoprophylaxis failure against mother-to-child HBV transmission
Source: BMC Infect Dis. 2019 Nov 21;19:985. doi: 10.1186/s12879-019-4624-9 (PMC6873716; doi:10.1186/s12879-019-4624-9)
Supplement: Supplementary file 1 — Additional file 1: Figure S1. The specificity of locked nucleic acid-based probe real-time PCR. (A) The wild-type plasmid (DNA level: 9.0 log copies/mL) was used for real-time PCR. The wild-type probe (FAM) detected the wild-type plasmid, but the G145A probe (HEX) did not detect the wild-type plasmid. (B) The G145A plasmid (DNA level: 9.0 log copies/mL) was sued for the real-time PCR. The Wild-type probe (FAM) did not detect the G145A plasmid, but the wild-type probe (HEX) did not detect the G145A-type plasmid. (PPTX 96 kb) [file 12879_2019_4624_MOESM1_ESM.pptx]

## Slide 1
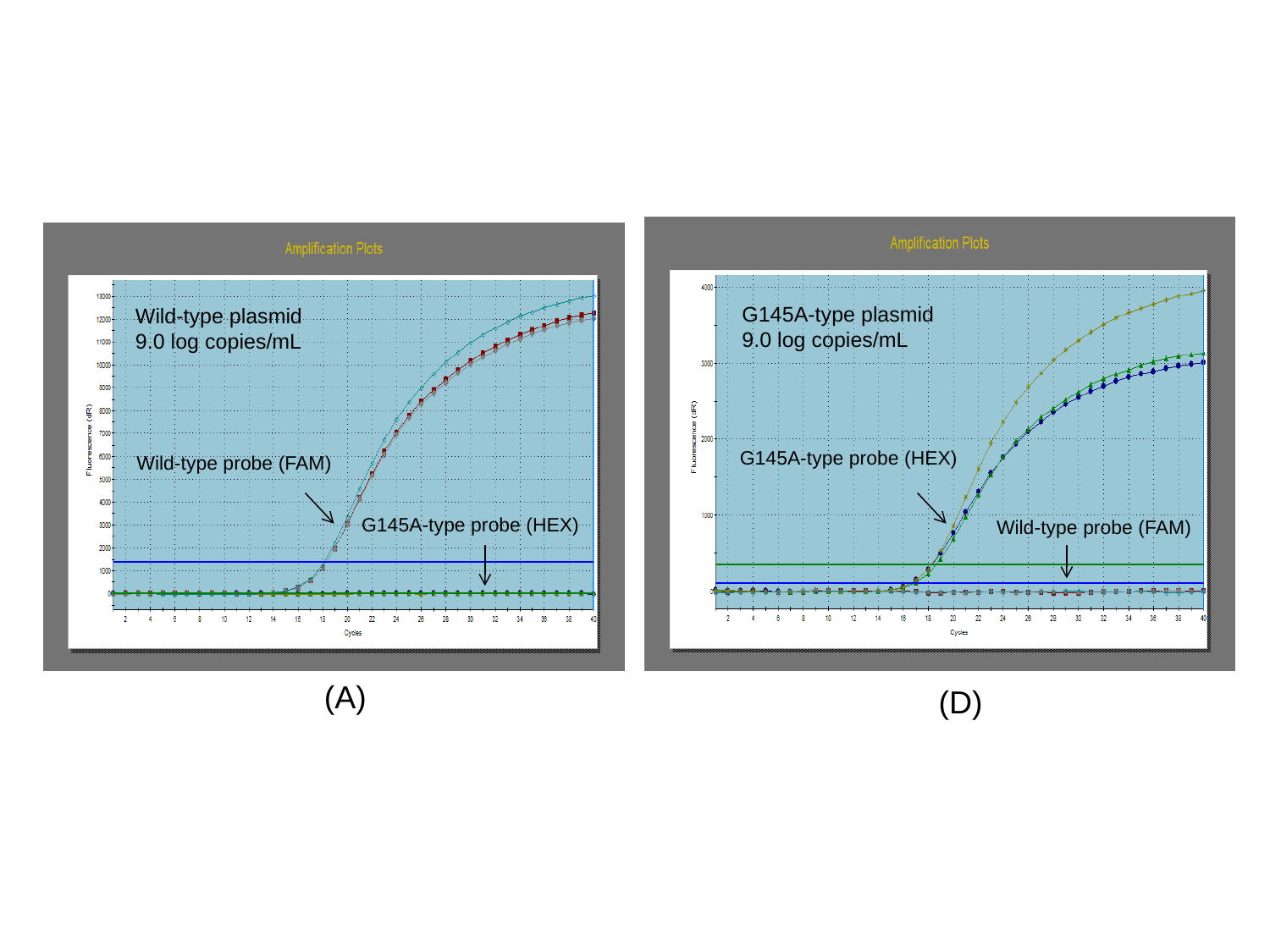

G145A-type plasmid
9.0 log copies/mL
Wild-type plasmid
9.0 log copies/mL
G145A-type probe (HEX)
Wild-type probe (FAM)
G145A-type probe (HEX)
Wild-type probe (FAM)
(A)
(D)
