# Supplementary material for: Deep sequencing of hepatitis B surface antigen gene in the preserved umbilical cords in immunoprophylaxis failure against mother-to-child HBV transmission
Source: BMC Infect Dis. 2019 Nov 21;19:985. doi: 10.1186/s12879-019-4624-9 (PMC6873716; doi:10.1186/s12879-019-4624-9)
Supplement: Supplementary file 2 — Additional file 2: Figure S2. The specificity of locked nucleic acid-based probe real-time PCR. Wild-type plasmid (DNA level: 8.2 log copies/mL) and G145A plasmid (DNA level: 8.2 log copies/mL) were mixed in this study. Population of G145R plasmid: (A) 100%, (B) 50%, (C) 25%, (D) 10%, and (E) 1%. (PPTX 153 kb) [file 12879_2019_4624_MOESM2_ESM.pptx]

## Slide 1
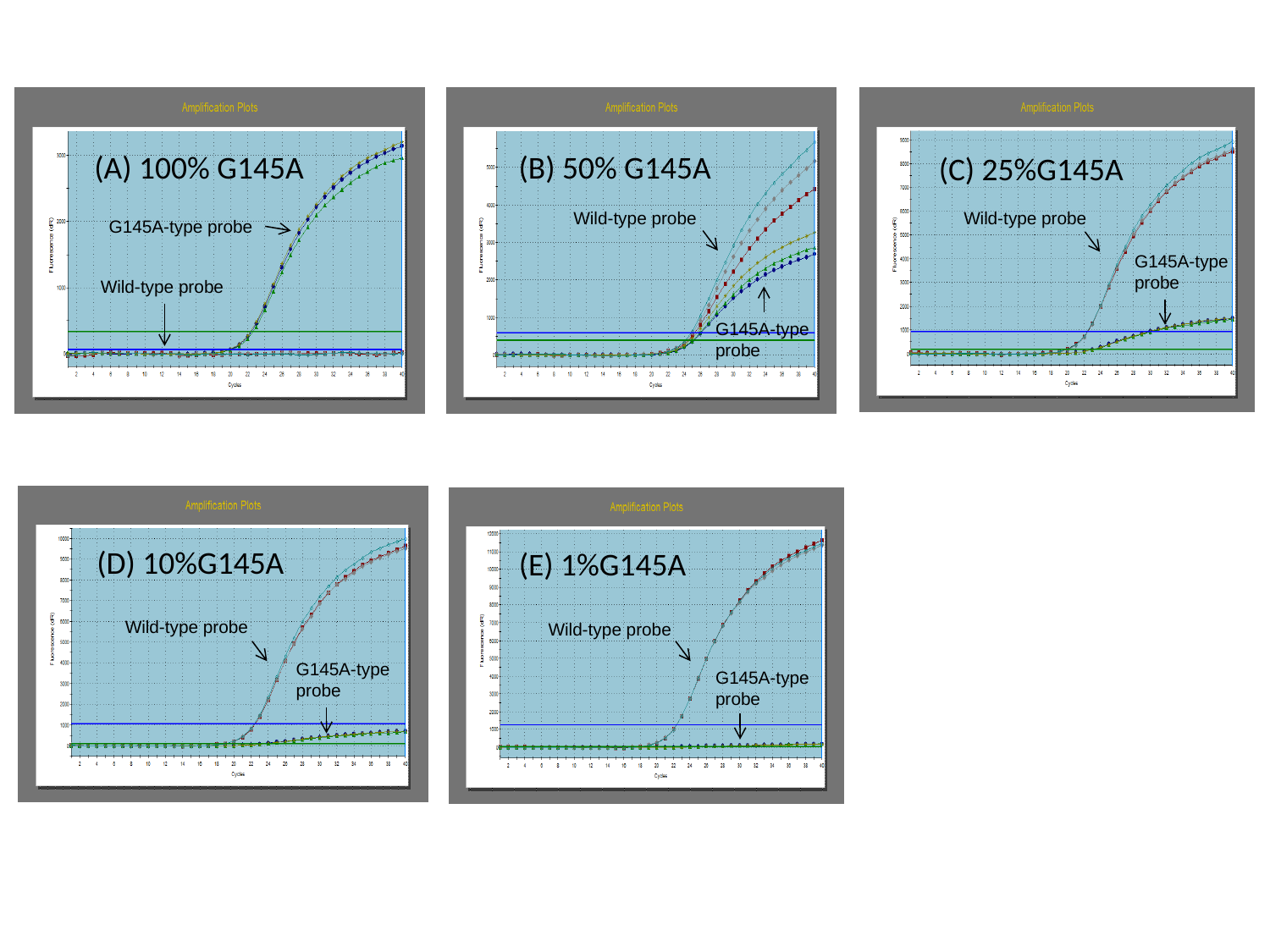

(A) 100% G145A
(B) 50% G145A
(C) 25%G145A
Wild-type probe
Wild-type probe
G145A-type probe
G145A-type probe
Wild-type probe
G145A-type probe
(D) 10%G145A
(E) 1%G145A
Wild-type probe
Wild-type probe
G145A-type probe
G145A-type probe
